# Using Teriparatide to Augment Healing in a Humeral Shaft Nonunion: A Case Report

**DOI:** 10.7759/cureus.39546

**Published:** 2023-05-26

**Authors:** Chaitanya S Puvvada, Jaithra S Marripaty

**Affiliations:** 1 General Surgery, Gayatri Vidya Parishad Institute of Health Care and Medical Technology, Visakhapatnam, IND

**Keywords:** parathyroid hormone (pth), nonunion fracture, delayed osseous union, proximal humerus fracture-dislocation, fracture, teriparatide

## Abstract

The occurrence of complications of fracture healing, such as delayed union and nonunion, is well known, but the use of pharmacotherapy for these delayed unions and nonunions has not been explored in detail. The authors describe a case of traumatic humeral shaft fracture successfully treated with once-daily administration of 20mcg of teriparatide for six months. The patient was a 22-year-old male who had been through a road traffic accident. The radiograph of the humerus shaft showed a fracture line and the displaced distal portion of the shaft of the humerus. Based on these features, the patient was diagnosed with a humeral shaft fracture. The patient underwent internal fixation with a dynamic compression plate. However, there were no signs of callus formation even after 12 weeks from the time of internal fixation. The patient was initiated with teriparatide administration and union was achieved after six months of a once-daily administration of teriparatide. Once-daily teriparatide treatment is shown to be beneficial for improving the healing of humeral shaft fractures showing delayed union.

## Introduction

Most humeral fracture unions normally occur in the course of three to four months. A transverse fracture as in this case is anticipated to require two times the duration for fracture union. Fracture nonunion and delayed union are complications that require surgical reintervention with an increased risk of postoperative complications. Teriparatide, a parathormone analog, available for the treatment of osteoporosis and hypo-parathryroidism, has been shown with limited evidence to have additional activity of fracture healing in delayed union and non-union, in a review and case reports [1].

Though the anabolic mechanisms of teriparatide in inducing callus formation in fracture nonunions are still not well known, many case reports and case series have described its efficacy in improving the healing of delayed and nonunions with very minimal or no side effects, making this drug an excellent conservative treatment of option for treating delayed and nonunions [2-13].

Parathyroid hormone is considered to be one of the key players in calcium homeostasis and calcium metabolism in the human body. Teriparatide a parathyroid hormone analog works by activating osteoblasts and inhibiting osteoblast apoptosis and as a result increasing their life span [14]. Teriparatide also works by increasing callus formation and improving the mechanical strength of bone at the fracture site [15]. In this article, we report a 22-year-old male with a humeral shaft fracture nonunion showed signs of callus formation and fracture union with the use of teriparatide.

## Case presentation

**Figure 1:**
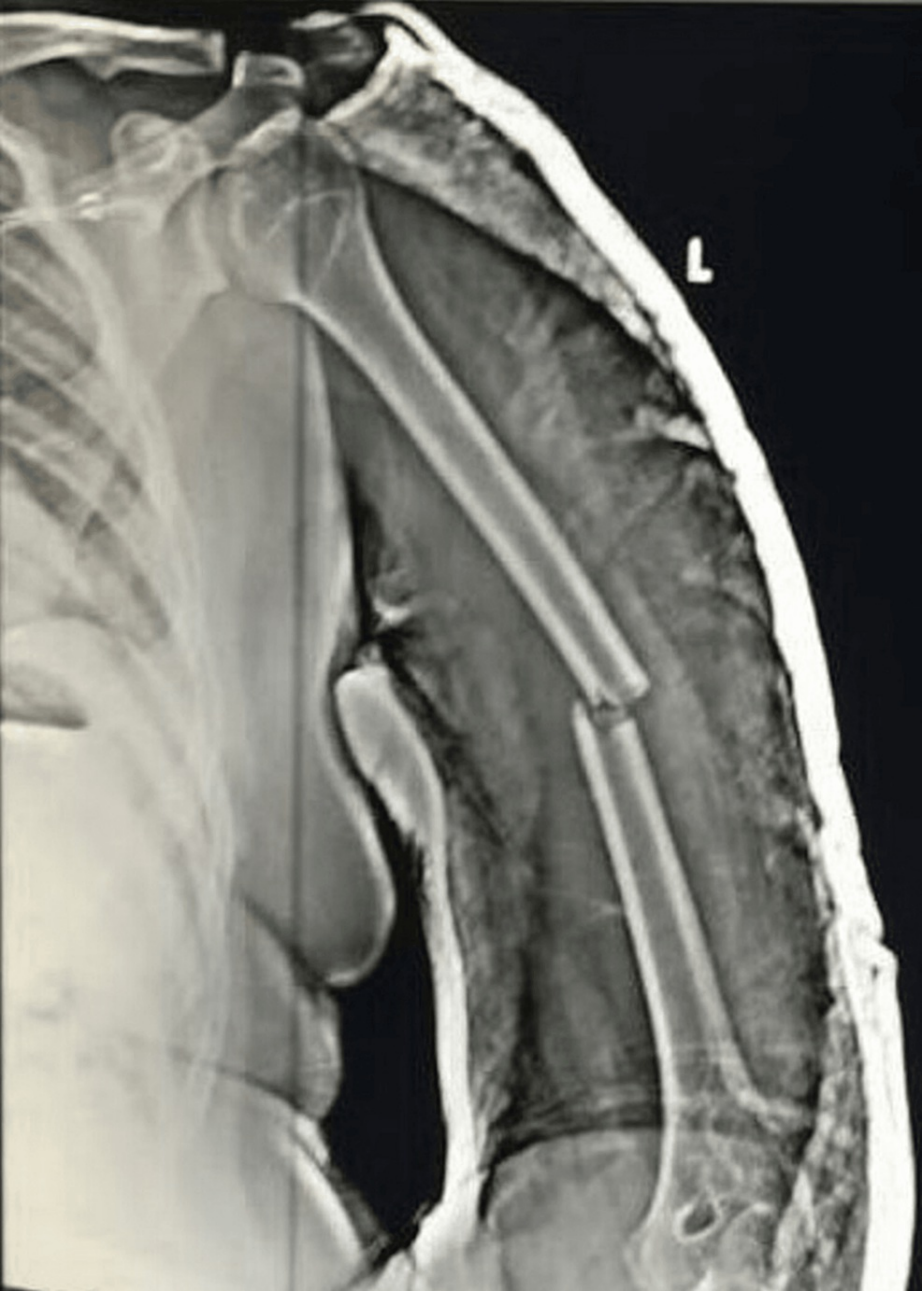
Radiograph taken immediately after reduction using a U slab

A 22-year-old male, right-hand dominant, was admitted to the hospital after he sustained an injury to the left arm from a road traffic accident. He presented with pain and mild swelling over his left arm. There was associated local tenderness and crepitus over the mid-arm region. No evidence of neurovascular compromise was found. A radiograph of the left arm revealed a displaced closed simple transverse fracture of the shaft of the humerus in its middle third. Closed reduction was done using a U slab as a preliminary care measure. The closed reduction obtained by the U slab can be seen in Figure 1.

**Figure 2:**
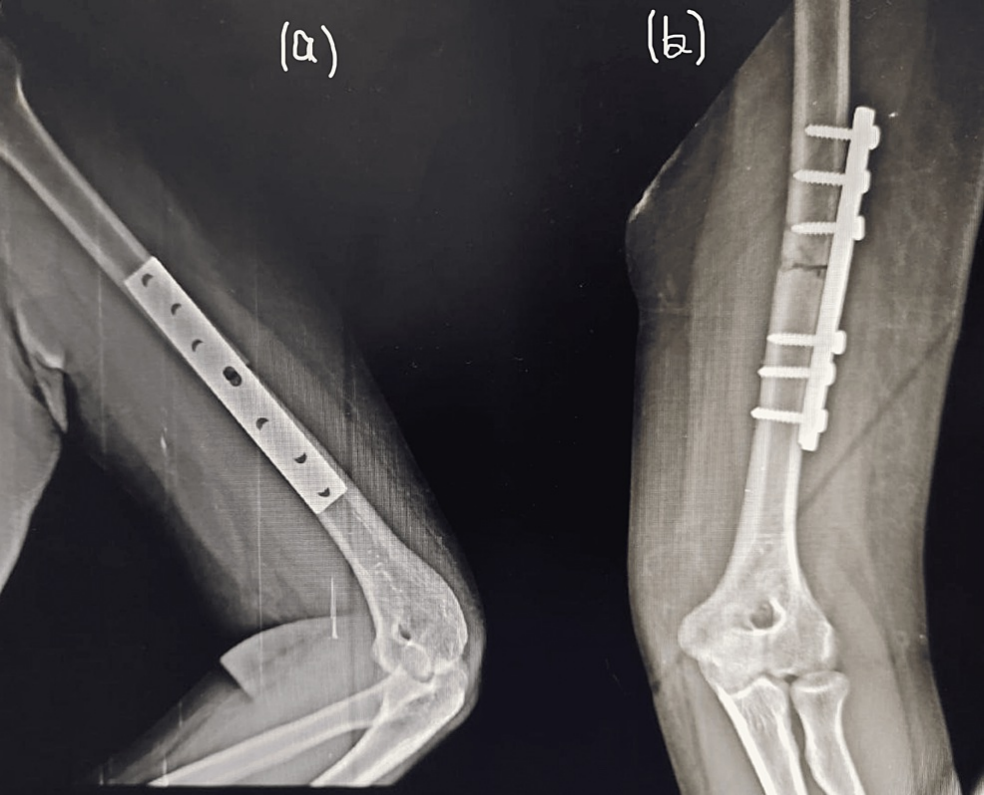
Radiograph showing fracture site six weeks after the internal fixation Left humerus and elbow joint (a) lateral view, (b) anteroposterior view

**Figure 3:**
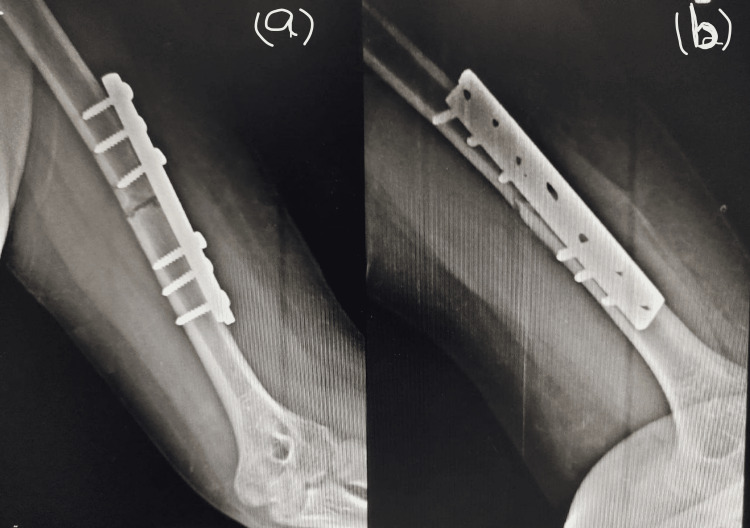
Radiograph showing the fracture site 11 weeks after the internal fixation Radiograph of left humerus (a) anteroposterior view, (b) lateral view

After considering the weight and the build of the patient a decision of open reduction was made. Open reduction and internal fixation with dynamic compression plate and screws via anterolateral approach was done after 24 hours from the time of fracture. Immediate radiograph taken after the surgical procedure showed well approximated bones. A follow-up radiograph taken after four weeks showed no callus formation. Another follow-up radiograph taken after eight weeks from the surgery also showed no progress and also revealed a slightly pushed out proximal portion of the dynamic compression plate (Figures 2a, 2b). A suspicion of lack of required compression force is made. A radiograph showing the fracture site 11 weeks after the internal fixation can be seen in Figures 3a, 3b. The patient was advised to add calcitonin nasal spray, as calcitonin is found to promote the cartilaginous phase of fracture healing by promoting an indirect positive response of vascular endothelium cells, monocytes and histiocytes. The calcitonin nasal spray showed no significant improvement. In a follow-up radiograph taken four weeks after starting the calcitonin nasal spray, no callus was formed.

**Figure 4:**
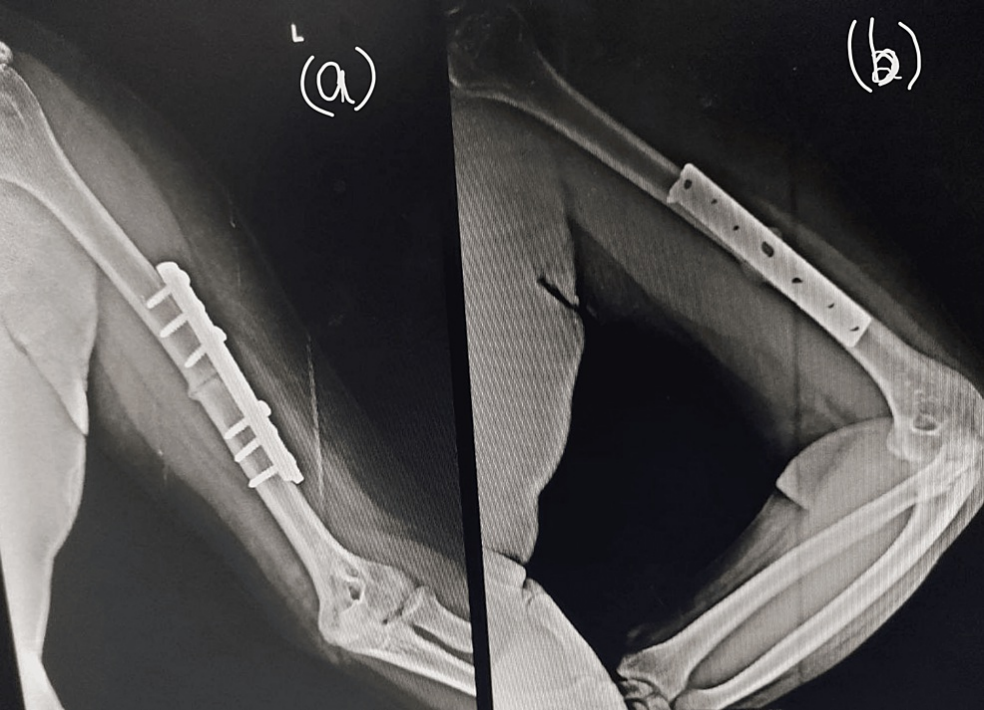
Callus formation seven weeks after initiation of teriparatide (19 weeks from the time of internal fixation) Radiograph showing left humerus (a) anteroposterior view, (b) lateral view

**Figure 5:**
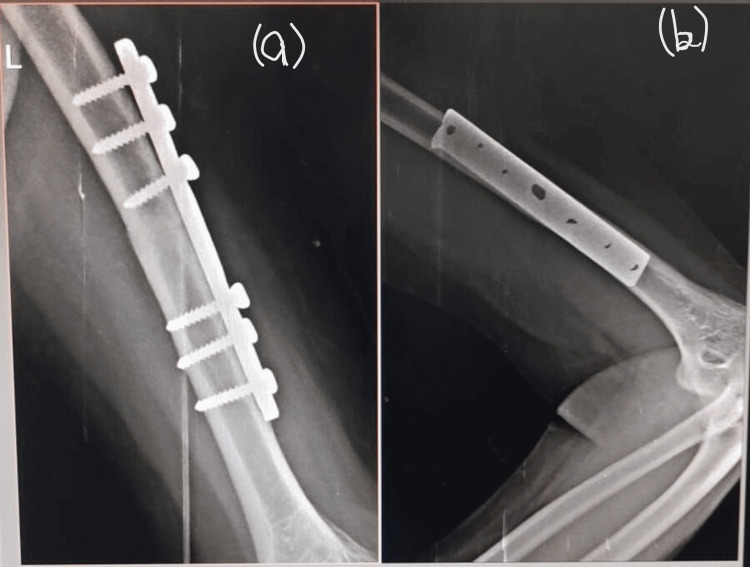
Radiograph showing fracture union, six months after the initiation of teriparatide (eight months from internal fixation) Radiograph of left humerus with plate in (a) anteroposterior view, (b) lateral view

The patient was advised teriparatide as a last resort before a bone marrow graft was considered. A daily dose of 20 micrograms of teriparatide was self-administered subcutaneously. A radiograph taken four weeks after the initiation of teriparatide showed the formation of a callus. He continued to use the same dose of teriparatide for another five months. Serial radiographs taken within this five-month duration (Figures 4a, 4b) showed a gradual closure of the fracture gap with an eventual complete union after six months from the start of teriparatide (Figures 5a, 5b). There were no observable side effects.

## Discussion

When nonunion occurs, treatment calls for a second surgical procedure to stabilize the bone and induce bone growth. These procedures include locked intramedullary nailing, dynamic compression plating, and external fixation using Ilizarov's principles. Autografting may also be necessary [8]. The anabolic drug teriparatide (rhPTH 1-34) is injected subcutaneously and may also be considered to promote bone healing. It has been approved for the treatment of glucocorticoid-induced osteoporosis as well as osteoporosis in men and postmenopausal women who are at high risk of fractures. The activation of the osteoblast, which results in a net increase in both cancellous and cortical bone, gives it its anabolic action, which enhances bone architecture [9]. Undoubtedly, a second intervention will be required, which is not without dangers and potential consequences and raises healthcare expenditures. Nonunion is a serious issue for the patient and has a significant impact on his quality of life [10]. Any remedy that can address this issue should therefore be used.

Our patient's fracture was initially treated with a U-shaped plaster and reduced, but due to the lack of an adequate reduction, an internal reduction using dynamic compression plating was performed using a dynamic compression plate and screws with a 2.5-mm diameter that were inserted retrogradely. As long as the plate stays in opposition, this method achieves a sufficient level of stability. In the week after surgery, an arm sling was used. Further clinical evaluations revealed radiographic nonunion signs like resorption of bone margins, absence of bone bridging, and a gap between fragments, which likely resulted from an unstable internal reduction. Other than using NSAIDs briefly, no additional clinical risk factors were discovered to support nonunion. There is considerable debate concerning the role of NSAIDs in delaying the union process in humans, and some reports advise avoiding using them in the immediate aftermath of a fracture [15].

Many case reports and case series describe the efficacy of teriparatide in the conservative management of fracture nonunions and delayed unions. Few prospective studies on this aspect of teriparatide showed significant positive results.

A prospective study done by Almirol et al. showed a positive result. The teriparatide-treated group showed a greater tibia cortical area and thickness compared to the placebo-treated group as early as eight weeks of treatment [16]. A prospective study done by Aspenberg et al. showed a positive result. Clinically approved teriparatide 20 µg dose significantly shortened the median time to healing compared with placebo treatment. They found a positive effect of teriparatide 20 µg but not 40 µg dose of teriparatide. 20 µg had a highly significant effect on reducing the median time to healing compared with a placebo [17]. A prospective study done by Bhandari et al. showed a positive result [18]. A prospective study done by Saraf et al. showed a positive result for delayed union fractures, administration of teriparatide was associated with an improved healing time compared with the placebo treated group [19]. A prospective study done by Kastirr et al. showed a positive result, after an average of 4.1 ± 1.5 (2-6) months after PTH treatment, 30 of the 32 patients experienced a stable osseous consolidation of the nonunion and regained full, pain-free weight-bearing capacity of the fractured extremity. The mean time between the initial fracture and the start of PTH treatment was 24.3 ± 17.8 months (9-84) [20].

## Conclusions

In this case report, we present an atrophic nonunion of humeral shaft fracture, the humeral shaft atrophic nonunion in this case was not treated in any way that would have slowed its course, and a direct link was found between the drug and healing. This case of diaphyseal nonunion in long bones was treated with teriparatide; given that teriparatide acts differently on the trabecular and cortical tissues, this finding raises the possibility that teriparatide might speed up the healing process in nonunions involving this kind of bone. The authors are cognizant of the fact that this is just one case report and that future research using a scientific methodology must be carried out in order to evaluate the effectiveness of teriparatide in fracture healing in both types of bone.
